# miR-132/212 Impairs Cardiomyocytes Contractility in the Failing Heart by Suppressing SERCA2a

**DOI:** 10.3389/fcvm.2021.592362

**Published:** 2021-03-19

**Authors:** Zhiyong Lei, Christine Wahlquist, Hamid el Azzouzi, Janine C. Deddens, Diederik Kuster, Alain van Mil, Agustin Rojas-Munoz, Manon M. Huibers, Mark Mercola, Roel de Weger, Jolanda Van der Velden, Junjie Xiao, Pieter A. Doevendans, Joost P. G. Sluijter

**Affiliations:** ^1^Experimental Cardiology Laboratory, Division Heart and Lungs, Department of Cardiology, University Medical Center Utrecht, Utrecht, Netherlands; ^2^Division Lab, Central Diagnosis Laboratory Research, University Medical Center Utrecht, Utrecht, Netherlands; ^3^Department of Medicine and Cardiovascular Institute, Stanford University School of Medicine, Stanford Cardiovascular Institute, Stanford, CA, United States; ^4^Department of Physiology, Amsterdam University Medical Center, Vrije Universiteit, Amsterdam Cardiovascular Sciences, Amsterdam, Netherlands; ^5^University Medical Center Utrecht Regenerative Medicine Center, Circulatory Health Laboratory, University Utrecht, University Medical Center Utrecht, Utrecht, Netherlands; ^6^Department of Genetics, Division Laboratories, Pharmacy and Biomedical Genetics, University Medical Center Utrecht, Utrecht, Netherlands; ^7^Regeneration and Ageing Lab, School of Life Science, Shanghai University, Shanghai, China; ^8^School of Medicine, Shanghai University, Shanghai, China; ^9^Netherlands Heart Institute, Utrecht, Netherlands; ^10^Central Military Hospital Utrecht, Utrecht, Netherlands

**Keywords:** miR-132/212 family, cardiac contractility, heart failure, myocardial infarction, knockout mice

## Abstract

Compromised cardiac function is a hallmark for heart failure, mostly appearing as decreased contractile capacity due to dysregulated calcium handling. Unfortunately, the underlying mechanism causing impaired calcium handling is still not fully understood. Previously the miR-132/212 family was identified as a regulator of cardiac function in the failing mouse heart, and pharmaceutically inhibition of miR-132 is beneficial for heart failure. In this study, we further investigated the molecular mechanisms of miR-132/212 in modulating cardiomyocyte contractility in the context of the pathological progression of heart failure. We found that upregulated miR-132/212 expressions in all examined hypertrophic heart failure mice models. The overexpression of miR-132/212 prolongs calcium decay in isolated neonatal rat cardiomyocytes, whereas cardiomyocytes isolated from miR-132/212 KO mice display enhanced contractility in comparison to wild type controls. In response to chronic pressure-overload, miR-132/212 KO mice exhibited a blunted deterioration of cardiac function. Using a combination of biochemical approaches and *in vitro* assays, we confirmed that miR-132/212 regulates SERCA2a by targeting the 3′-end untranslated region of SERCA2a. Additionally, we also confirmed PTEN as a direct target of miR-132/212 and potentially participates in the cardiac response to miR132/212. In end-stage heart failure patients, miR-132/212 is upregulated and correlates with reduced SERCA2a expression. The up-regulation of miR-132/212 in heart failure impairs cardiac contractile function by targeting SERCA2a, suggesting that pharmaceutical inhibition of miR-132/212 might be a promising therapeutic approach to promote cardiac function in heart failure patients.

## Introduction

Intracellular calcium and cardiomyocyte contractility act in a well-concerted manner, known as excitation and contraction coupling (1). In a normal heart contraction-relaxation cycle, the cardiac action potential triggers calcium entry into the cell *via* L-type calcium channels. This small increase of cytosolic calcium then stimulates a bulk release of calcium from the sarcoplasmic reticulum (SR) into the cytosol *via* the Ryanodine receptor (RYR2) (1). The elevated cytosolic calcium concentration stimulates the contractile machinery of the myofilaments. After contraction, for relaxation and diastolic filling to occur, cytosolic calcium has to be removed to turn off the contractile machinery. In mammalian cells, cytosolic calcium undergoes re-uptake by the SR mainly *via* the sarcoplasmic-endoplasmic reticulum Ca^2+^ ATPase 2 (SERCA2) (2). Thus, the activity of SERCA2 is critical to maintaining the regular heart contraction-relaxation cycles. In the failing heart, however, cardiomyocytes cannot maintain a proper excitation and contraction coupling, which is still not fully understood but often associated with a decreased SERCA2a expression, which results in impaired re-uptake of Ca^2+^ into the SR (3, 4). People have made significant efforts to rescue cardiac contractile function through restoration of the expression of SERCA2 directly (5–7) or through indirect enhancement of its activity (8). Unfortunately, the results from the Phase 2b CUPID2 trial failed to show any significant treatment effect, probably related to ineffective normalization of SERCA2 levels under the conditions tested. To develop an effective pharmaceutical treatment to restore SERCA2 expression level, understanding the underlying mechanism of the down-regulation of SERCA2 in failing hearts is still warranted.

MicroRNAs (miRNAs) are small non-coding RNAs that play an essential role in regulating cardiac development and maintenance of cardiac function. Dysregulation of miRNAs has been associated with the progressive pathological development of several cardiac diseases (9), and pharmaceutical targeting of miRNAs is beneficial in slowing down disease progression (10). MiRNAs can modulate protein expression through either the degradation or translational inhibition of their target messenger RNA (mRNA) molecules. As miRNAs exert an inhibitory effect on gene expression and some miRNAs are upregulated in the failing heart (11), upregulated miRNAs may suppress SERCA2 expression. Removing or blocking those miRNAs might be a way to restore SERCA2 expression levels. To identify those miRNAs, we previously screened for miRNAs, which can suppress SERCA2 expression and regulate cardiomyocyte calcium decay kinetics (12) and found miRNA-25 as a regulator of SERCA2. In this screen, the miR-132/212 cluster is as one of the top hits (12). This miR-132/212 family regulates cardiac function in the failing mouse heart, and pharmaceutically inhibition of miR-132 is beneficial for heart failure (11, 13, 14). However, their specific role in cardiac contractile regulation and development of heart failure remains to be well-defined.

In this study, we reveal that the miR-132/212 family is involved in the regulation of calcium handling and that the up-regulation of these miRNAs may impair cardiac contractility in failing hearts. We show that neonatal cardiomyocytes overexpressing miR-132/212 have a prolonged calcium decay *in vitro*, and cardiomyocytes isolated from miR-132/212 KO mice exhibit enhanced contractility *ex vivo*. Moreover, the miR-132/212 KO mice are protective against pressure overload-induced cardiac dysfunction. These observations suggest that miR-132/212 may be a promising therapeutic target to promote cardiac function in heart failure patients.

## Materials and Methods

### Generation and Genotyping of miR-132/212 KO Mice

The generation of miR-132/212 KO mice has been described previously (15, 16). Briefly, the floxed miR-212-miR132 mice were generated by gene targeting in an ES cell line from C57BL/6N, then crossed with a Cre delete line to remove miR-212-miR-132 region. The result miR-212-132^−/−^ line is in C57BL/6J. The animal experiment was all carried out using this strain with age and sex-matched C57BL/6J mice as wildtype (WT) control. For genotyping, genomic DNA was extracted from ear clippings using the genomic DNA isolation kit (Sigma, Cat. XNATS). PCR was done with the GC-Rich PCR kit (TAKARA, Cat. RR002C) with miR-132/212 primers, as shown in Supplementary Material. PCR products were separated on 1% agarose gel: WT gave a band at 1,076 bp and the KO at 392 bp.

### Human Cardiac Tissue

The use of human cardiac tissue was approved for research purposes by the Medical Ethics Committee of the University Medical Center Utrecht, The Netherlands and the study was performed conform to the declaration of Helsinki. The left ventricular wall of patients with end-stage heart failure was used while the left ventricular tissue from refused donor hearts was used as healthy controls.

### TAC Mouse Model and Echocardiography

This study was approved by the Animal Ethical Experimentation Committee (DEC. 2013.II.02.019, Utrecht University) and was carried out under the Guide for the Care and Use of Laboratory Animals. Transverse aorta constriction was performed as previously described (17). In brief, 12 weeks old miR-132/212 KO mice or their WT control litters were subjected to TAC or sham surgery. Surgical procedures were performed under sterile conditions with breathing pump and mice were anesthetized with fentanyl (0.05 mg/kg), midazolam (5 mg/kg), and medetomidine (0.5 mg/kg) by intraperitoneal injection. After exposing the transverse aorta, a 27 gauge constriction was made between the first and second branches of the aortic as previously described. After closure, mice recovered upon atipamezole (2.5 mg/kg) and flumazenil (0.5 mg/kg). After surgery, Temgesic (0.1 mg/kg) was given as a painkiller every 8 h for 2 days. Sham surgery was conduction in the same way without gauge constriction. Echocardiography and cardiac function testing were performed both before and 7 weeks following TAC with the Vevo® 2100 System (Visualsonics) and analyzed with Vevo2100-1.6.0 (Visualsonics) software as previously described. During the measurement, mice were under anesthesia with isoflurane and their body temperature was maintained at 37°C. Two mice dropout 1 day after surgery where were excluded later analysis. To terminate the mice, mice were anesthetized overdose anesthesia with fentanyl (0.1 mg/kg), midazolam (10 mg/kg), and medetomidine (1 mg/kg) by intraperitoneal injection.

### *Ex vivo* Cardiomyocyte Calcium Imaging and Sarcomere Shortening Measurement

For cardiomyocytes isolation, mice were first injected with 100 IU/kg heparin 20 min before termination. Then mice are terminated by overdose anesthesia as described above. Freshly explanted mice hearts were cannulated and perfused with perfusion-buffer (11.3 mM NaCl, 0.47 mM KCl, 60 mM KH_2_PO_4_, 60 mM Na_2_HPO_4_, 120 mM MgSO_4_, 1.2 mM NaHCO_3_, 1 mM KHCO_3_, 1 mM HEPES, and 3 mM Taurine) for 10 min at 37°C, then switched to an enzyme solution (1x perfusion buffer, 5.5 mM glucose and 5.0 mM 2, 3-Butanedione monoxide with Roche's Librase) for about 7 min. The hearts were then removed from the perfusion device and placed on petri-dish with stop buffer. The left ventricle was removed and cut into pieces before being gently triturated with a plastic Pasteur pipet to release single cardiomyocytes. After centrifuging at 100 g for 1 min, myocytes were resuspended in 1 mM CaCl_2_ with the final concentration gradually adjusted to 2 mM. Cells were loaded with Fura-4 at 1 μM in HEPES buffer for 30 min. After washing, cells were placed under the IonOptix imaging system, paced at 1 Hz, 20 mV at 1 ml/min continuous flow at 37°C. Cells with normal cardiomyocyte morphology (rod-shaped, clear striations) and sarcomeres (without bubs or signs of spontaneous contraction) are recorded. Approximately 30–50 cells per heart were recorded and analyzed. Data were analyzed by Ionwizard 6.0. Calcium.

### miRNA *in situ* Hybridization

The detailed protocol for miRNA *in situ* hybridization has been described previously (18). Briefly, 10 mm sections were fixed with 4% PFA for 10 min before proteinase K treatment (5 mg/ml) for 10 min. Subsequently, sections were re-fixed with 0.16 M EDC (PI-22980 Thermo Fisher) in 0.13 M 1-methylimidazole for 1 h at 37°C, then acetylated for 10 min at room temperature. After incubation for 1 h with urea-based hybridization buffer, DIG-labeled miRCURY LNA miRNA detection probes (Exiqon) for miR-132 (38031-15), negative control mir-159 (99003-15), and positive control U6 (99002-15) at a final concentration of 25 mM were applied to the sections overnight. After washing, sections were subsequently blocked for 1 h before overnight incubation with anti-DIG alkaline phosphatase antibody (1:1,500, Roche), sections were incubated with levamisole solution (X302130, DAKO) To block endogenous alkaline phosphatase activity. NBT/BCIP (K059811, DAKO) substrate was then added for visualization. Antigen retrieval was performed using a pressure cooker with citrate buffer pH6.0 before immunohistochemistry staining with other cellular markers. The Blood vessels in mouse tissue were stained with lectin BS-1 (1:200, Sigma). Nuclei were stained with Hoechst 33342 (Life Technologies). Cardiomyocytes were stained with Troponin (ab47003, Abcam). Samples from three different mice each group used for analysis and staining was repeated at least once. Images were taken by Zeiss LSM710 and analyzed using Zen 2012 (Zeiss).

### Mouse Heart Failure Models

To examine the expression of miR-132/212 in different heart failure model, total RNA was extract from healthy heart, 7 weeks transverse aortic constriction (TAC)-operated mice heart (17), Angiotensin-II infused mouse heart (19), Calcineurin overexpressing mouse heart (20), and muscle LIM protein (MLP) KO mice heart (21).

### RNA Isolation and RT-PCR

For miRNA quantification, RNA was extracted with Tripure (Roche) following manufacture instruction, generation of cDNA and qPCR was conducted with the Taqman® miRNA Reverse Transcription Kit and Taqman® miRNA Assay Kit using 2 ng total RNA as input. RNU6 was used as internal control for normalization. For gene expression quantification, RNA was prepared with the RNA isolation kit (Macherey-Nagel, Nucleospin RNA kit), transcribed to cDNA using the iScript cDNA Synthesis Kit (Bio-Rad) according to manufacturer instructions, and quantitative real-time qPCR was performed on an RT-PCR system (Bio-Rad) using 500 ng per reaction (22). The expression of genes was normalized by the expression of GAPDH. All the primers used for qPCR analysis are listed in Supplementary Table 1.

### Immunofluorescent Staining

Sections were fixed with cold methanol and subsequently blocked with 10% normal goat serum plus 2% BSA in TBST, containing 0.1% Tween-20. Sections were then incubated with primary antibodies diluted in 0.5% BSA in TBST overnight at 4°C. Images were taken by Zeiss LSM700 and analyzed using ZEN 2012 software (Zeiss). Antibodies and concentrations used in this study are listed in Supplementary Table 2.

### Western Blotting

Cells or cardiac tissue were lysed with lysis buffer (50 mm Tris-HCL pH 7.4, 100 mm NaCl, 1% NP40, 0.1% SDS, 0.5% sodium deoxycholate) supplemented with 1x protease/phosphatase inhibitor cocktail (Cell Signaling, #5872). Protein concentrations were measured with BCA protein assay kit (Thermo Scientific, 23227), 5 μg cell lysate or 20 μg tissue lysate was loaded on Nupage bis-tris Precast gels (Life Technologies), and transferred to PVDF membrane with iblot 2 Western blotting system (Life Technologies), according to manufacturer instructions. Membranes were first blocked with 5% blotting grade blocker (Bio-Rad #170-6404). After washing, primary antibodies were diluted in 5% TBST and applied to the membrane overnight at 4C. After washing, appropriate horseradish peroxidase HRP-conjugated secondary antibodies were used for enhanced chemiluminescent (ECL) detection (Sigma). All the antibodies used in these studies and their dilution are listed in Supplementary Table 2.

### Cell Culture, Transfection, and Calcium Imaging in NRCM

For calcium imaging in Neonatal Rat CardioMyocytes (NRCMs), NRCMs were isolated from neonatal Sprague Dawley rat within 3 days after born using Pierce™ Primary Cardiomyocyte Isolation Kit following the provider's instructions. NRCM cells were transfected with either 20 nmol/L mirVana miRNA mimic negative control (4464085), hsa-miR-132-3p mimics (MC10166), hsa-miR-212-3p mimics (MC10340), mirVana miRNA inhibitor negative control1 (4464077), hsa-miR-132-3p inhibitor (AM10166), hsa-miR-212-3p inhibitor (AM10340) using RNAiMAX (Life Technologies). After 6 h, media was replaced with fresh Claycomb media and cells maintained for 48 h before harvest. Calcium imaging in the NRCMs were performed with automated calcium kinetic imaging method which has been described in our previous paper (23).

### 3′-Untranslated Region (3′-UTR) Reporter Generation and Luciferase Assay

The potential miR-132/212 binding sites on 3′UTR of SERCA are predicted by Targetscan (24). A 0.8 kb fragment of SERCA2a 3′-untranslated region (3′-UTR) was cloned into the pMIR-REPORT Luciferase vector (Ambion), as described previously (22). Mutations in the seed-region were generated by the Q5 Site-Directed Mutagenesis kit (New England Biolabs) as indicated in **Figure 3A**. All the primers used for cloning and mutagenesis are listed in Supplementary Table 1. HEK293 cells were co-transfected with 200 ng of pMIR-REPORT-3′-UTR Luciferase vectors, or one of the mutated vectors, and a pMIR-REPORT β-gal control plasmid to normalize for transfection efficiency together with 25 nmol/L miRNA mimic controls, miR-132 mimics, or miR-212 mimics with Lipofectamine 2000 (Life Technologies) to test suppression efficiency of miR-132 and 212 on luciferase activity. Luciferase and β-galactosidase activity was measured after 48 h with the Luciferase Assay System and β-galactosidase Enzyme Assay System (both from Promega), respectively, as previously described (25).

### Statistical Analysis

Data were analyzed using Graphpad Prism 6 and comparisons were performed with a *t*-test or paired *t*-test between two groups and ANOVA with Bonferroni correction for multiple comparisons. Data are presented as mean ± SEM. ^*^p < 0.05 is considered as significantly different.

## Results

### miR-132/212 Regulate Cardiomyocytes Contractile Function *via* SERCA2

**Figure 1:**
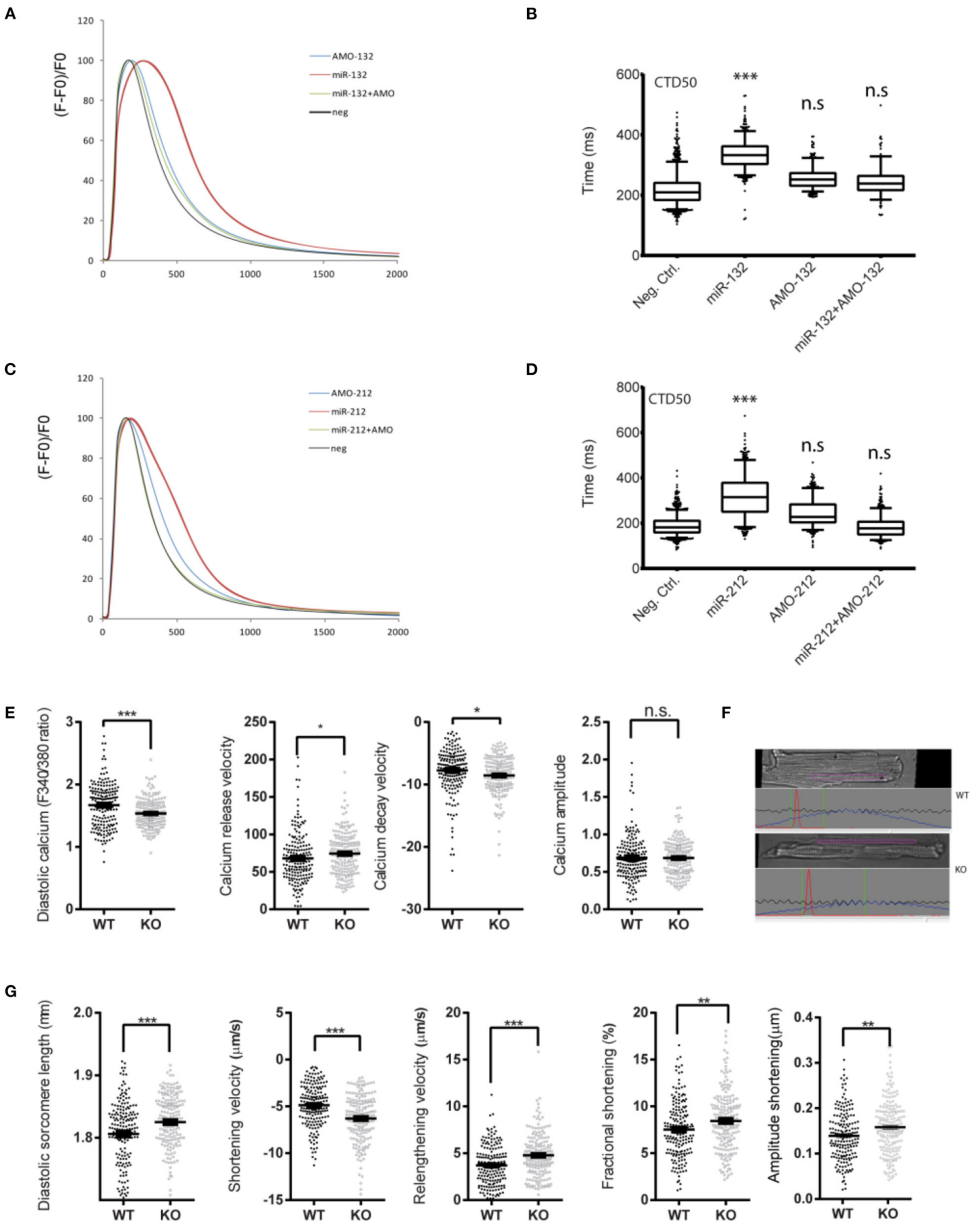
Effect of loss or gain of function of miR-132/212 on cardiomyocytes contractility. **(A)** Calcium kinetics after overexpression and inhibition of miR-132 on as measured by Fluo-4 intensity. **(B)** The de- cay time CTD50 after overexpression and inhibition of miR-132. **(C)** Calcium kinetics after overexpression and inhibition of miR-212 on as measured by Fluo-4 intensity. **(D)** The decay time CTD50 after overex-pression and inhibition of miR-212. **(E)** Diastolic calcium (F340/380), calcium release velocity, calcium decay velocity, and the calcium amplitude of the Fura-2 trace in cardiomyocytes isolated from WT (*n* = 6) and KO (*n* = 6) mice. **(F)** Representative image sarcomere shortening analysis of cardiomyocytes isolated from WT and KO mice. **(G)** The diastolic sarcomere length, shortening velocity, relengthening velocity, fractional shortening, and the amplitude shortening of cardiomyocytes isolated from WT (*n* = 6) and KO (*n* = 6) mice. CTD50: 50% Calcium transient durations, AMO, antimiR oligonucleotides. *p < 0.05,**p < 0.01, ***p < 0.001. n.s indicates not statistical significant different.

**Figure 2:**
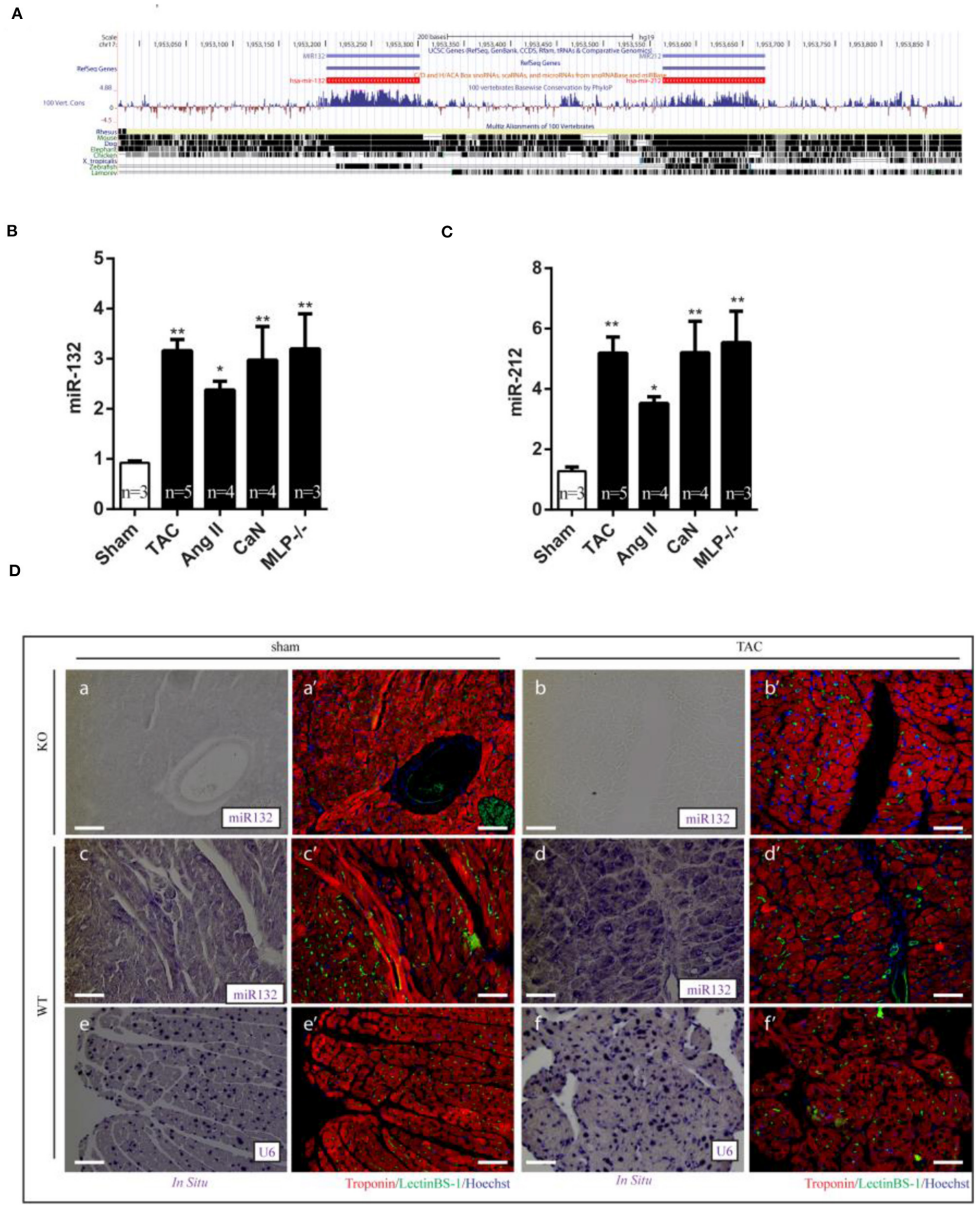
Dysregulation of miR-132/212 expression in left ventricle from hypertrophic mouse models and heart failure patients. **(A)** The location of miR-132/212 loci in human genome (chr17:1,953,129-1,953,734) and conservation of miR-132 and miR-212 between different species (27). **(B)** The relative expression of miR-132 in the left ventricle from Sham, TAC, Ang II, can, MLP-/- induced hypertrophic mouse hearts. RNU6 is used as internal control. **(C)** The relative expression of miR-212 in the left ventricle from Sham, TAC, Ang II, can, MLP-/- induced hypertrophic mouse hearts. RNU6 is used as internal control. **(D)** *in situ* detection of miR-132 in Sham-operated miR-132/212 KO mice (a,a'), TAC (b,b') in sham-operated wildtype mice (c,c'), TAC (d,d'), *in situ* detection of U6 served as a control in sham-operated wildtype mice (e,e'), TAC (f,f'). Immunofluorescent staining for Lectin BS-1 for endothelial cells in green, Troponin I for viable cardiomyocytes in red, nuclei in blue. *p < 0.05, **p < 0.01, ***p < 0.001. n.s indicates not statistical significantly different.

To screen for miRNAs that can suppress SERCA2 expression, we previously identified several microRNAs which suppress GFP expression of the GFP/SERCA2-3′UTR reporter. One of the identified hits is the miR-132/212 family, a highly conserved microRNA family among different species (**Figure 2A**), which inhibits the GFP/SERCA2a-3′-UTR expression in HL-1 cells (12). In this follow-up study, we aimed to confirm and further explore the role of miR-132/212 in the regulation of SERCA expression. First, we tested the effect of miR-132/212 overexpression on cardiomyocyte contractility in primary isolated neonatal rat cardiomyocytes (RNCM). Using automated calcium kinetic imaging (12, 26), we found that miR-132 and miR-212 overexpression indeed significantly prolonged the decay of calcium transients (Figures 1A–D). This effect could be reversed by using microRNA inhibitors antimiR-132 or antimiR-212 (AMO), respectively (Figures 1A–D). Interestingly, we also observed a significantly slower Ca^2+^ influx in cardiomyocytes overexpressing miR-132 (time to peak) but not in the miR-212 overexpressing cells (Figures 1A,C). These results indicate that both miR-132 and miR-212 may regulate calcium re-uptake, thereby explaining the prolongation of calcium kinetics. Additionally, miR-132 but not -212 may also play a role in calcium release from the SR.

To further investigate the physiological role of miR-132/212 in the regulation of cardiomyocyte contractility, taking advantage of our miR-132/212 knockout (KO) mice (15), we isolated individual cardiomyocytes from these KO mice and their littermate wildtype (WT) controls. Using an IonOptix calcium and sarcomere shortening imaging system (Figure 1F), we simultaneously recorded the calcium kinetics and sarcomere shortening of these individual cardiomyocytes. In accordance with the prolonged calcium decay by overexpressing miR-132/212 in RNCM, the loss of miR-132/212 significantly enhanced cardiomyocyte contractility. This is indicated by the enhanced Ca^2+^-release and Ca^2+^-decay velocities of the calcium tracing in cells from the KO animals (Figure 1E), by the diastolic sarcomere length, the enhanced shortening velocity, the shortening amplitude, the fractional shortening and the re-lengthening velocity of isolated cells from the KO animals (Figure 1G). We also noticed that the KO animals have lower diastolic calcium, longer diastolic sarcomere length, which indicated less residual Ca^2+^, thus faster calcium re-uptake after contraction (Figure 1E).

### Upregulated miR-132/212 Expression in the Heart of Hypertrophic Mouse Models

Having demonstrated the role of miR-132/212 in regulating cardiomyocytes contractility, we determined the expression of this family in different physio- and pathological conditions. We monitored the expression of miR-132/212 in normal hearts and different rodent models for heart failure by RT-PCR. miR132/212 mRNA expression was compared between healthy, 7 weeks transverse aortic constriction (TAC)-operated (17), Angiotensin-II infused (19), Calcineurin overexpressing (20), and muscle LIM protein (MLP) KO mice (21). Levels of both miR-132 and miR-212 were upregulated in all hypertrophic heart failure mouse models, indicating a potential common mechanism in these models (Figures 2B,C).

To further determine which cell types are expressing miR-132/212, we performed miRNA *in situ* hybridization. miR-132/212 localized mainly in cardiomyocytes (Figure 2D), with some detection also in large vessels (Figure 2D,d'). Consistent with the qPCR assay results (Figures 2B,C), miR-132 was found to be significantly upregulated in cardiomyocytes from TAC animals (Figure 2D,d').

### SERCA2a and PTEN as Potential Targets of miR-132/212

**Figure 3:**
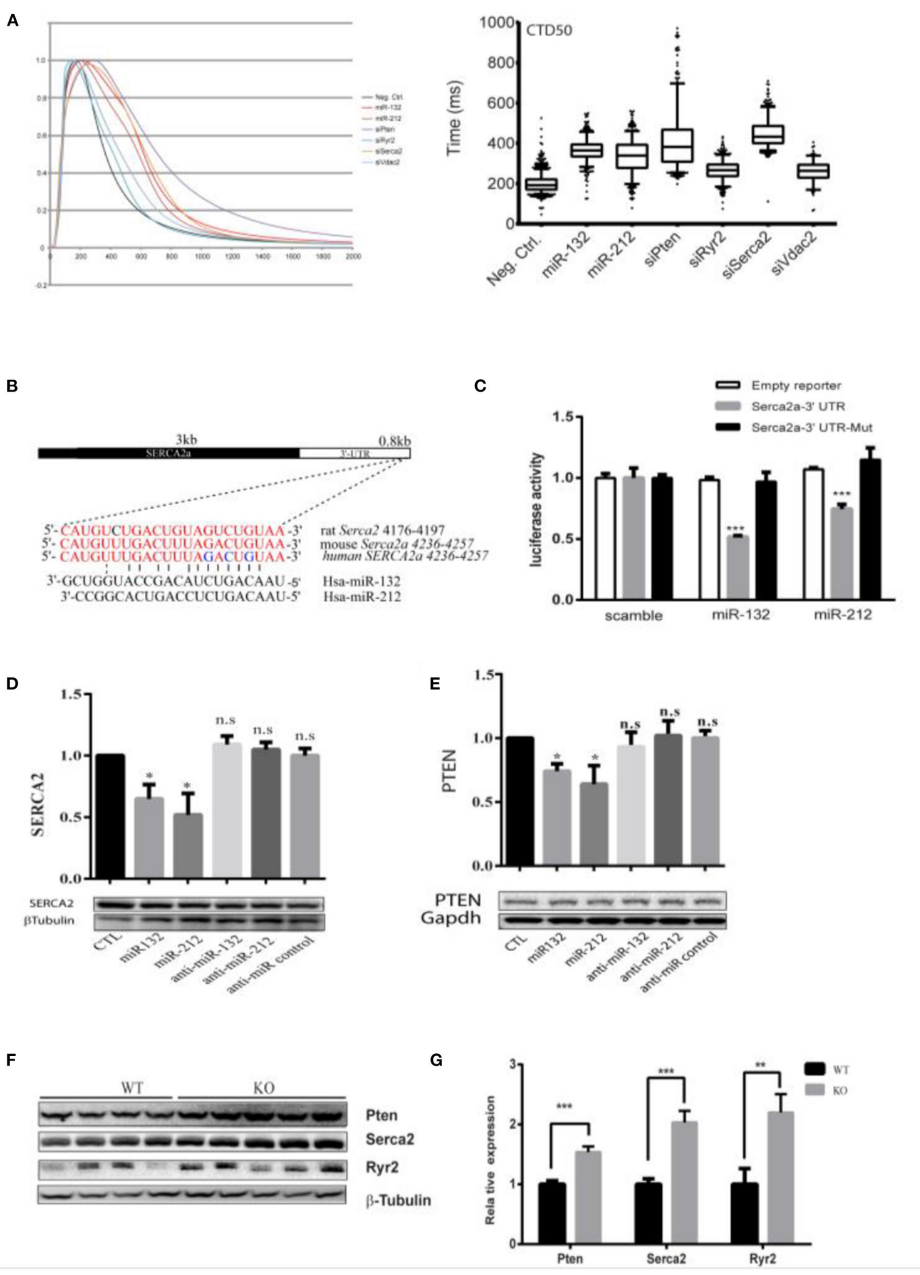
Serca2a and PTEN as targets of miR-132/212. **(A)** The effect of knockdown predicted targets of miR-132/212 PTEN, Ryr2, VDAC2, and SERCA2 on calcium kinetics in RNCM as measured by Fluo-4 intensity. The decay time CTD50 of Fluo-4 signal after PTEN, RYR2, VDAC2, and SERCA2 knockdown and miR-132/212 overexpression. **(B)** Predicted miR-132/212 targeting site on 3′-UTR of SERCA2a, and site mutation on the 3′-UTR. **(C)** Luciferase activity assay of luciferase reporter with intact SERC2a-3′UTR or 3 nucleic acids mutated 3′-UTR after transfection of synthetic miRNA mimics for the scramble, miR-132 or miR-212 normalized by β-gal activity (*n* = 3). **(D)** Total endogenous Serca2 expression protein after transfection of scramble mimics (lane 1), miR-132 (lane 2), miR-212 (lane 3), antimiR-132 (lane 4), antimiR-212 (lane 5), and antimiR mimics (lane 6) by Western blot. Quantification of Serca2 expression corrected by β-Tubulin is plotted above (*n* = 3). **(E)** Total endogenous PTEN protein after transfection of scramble mimics (lane 1), miR-132 (lane 2), miR-212 (lane 3), antimiR-132 (lane 4), antimiR-212 (lane 5), and antimiR mimics (lane 6) by Western blot. Quantification of PTEN expression corrected by GAPDH is plotted above (*n* = 3). **(F)** PTEN, SERCA2, and Ryr2 protein level between WT and miR-132/212 Knockout mice by Western blot. **(G)** Quantification of PTEN, SERCA2, and Ryr2 protein in **(G)** corrected by β-Tubulin WT (*n* = 4), KO (*n* = 5). *p < 0.05,**p < 0.01, ***p < 0.001. n.s indicates not statistically significant different.

To explore the mechanism of miR-132/212 in regulating cardiomyocyte Ca^2+^ handling, we selected several potential miR-132/212 targets as predicted by Targetscan, namely SERCA2a, PTEN, and VDAC2 (Supplementary Figure 1). We hypothesized that knockdown of the targets of miR-132/212 should at least partially mimic the phenotype of miR-132/212 overexpression in prolongation of calcium decay. Therefore, we knocked down these potential targets by siRNA and compared them with miR-132/212 overexpression. We subsequently observed that knockdown of SERCA2a and PTEN could significantly prolong the calcium decay phase, which mimics the calcium kinetics trace of overexpression of miR-132/212 in RNCM (Figures 3A,B). Meanwhile, knockdown of Ryr2 and VDAC2 did not display such mimicry (Figures 3A,B). These results suggest that SERCA2a and PTEN might be the targets of miR-132/212 involved in calcium handling in RNCM.

Since PTEN is an established target of miR-132 by 3′-UTR luciferase reporter assays (28), we evaluated SERCA2a similarly. We observed that miR-132 and miR-212 significantly suppress the luciferase activity of the full-length SERCA2a 3′-UTR reporter but not of a mutated SERCA2 3′-UTR reporter (Figure 3C and Supplementary Figure 1B). Likewise, SERCA2a and PTEN protein expression was inhibited by transfection of miR-132 and miR-212 mimics in RNCMs (Figures 3D,E), although repression was mild. Additionally, also in our miR-132/212 KO animals, the expression of SERCA2 and PTEN in the heart are significantly increased (Figures 3F,G). Taking together, these results show that SERCA2 and PTEN can be regulated by miR-132/212.

### Loss of miR-132/212 Blunts the Pressure Overload-Induced Worsening of Cardiac Contractile Function

**Figure 4:**
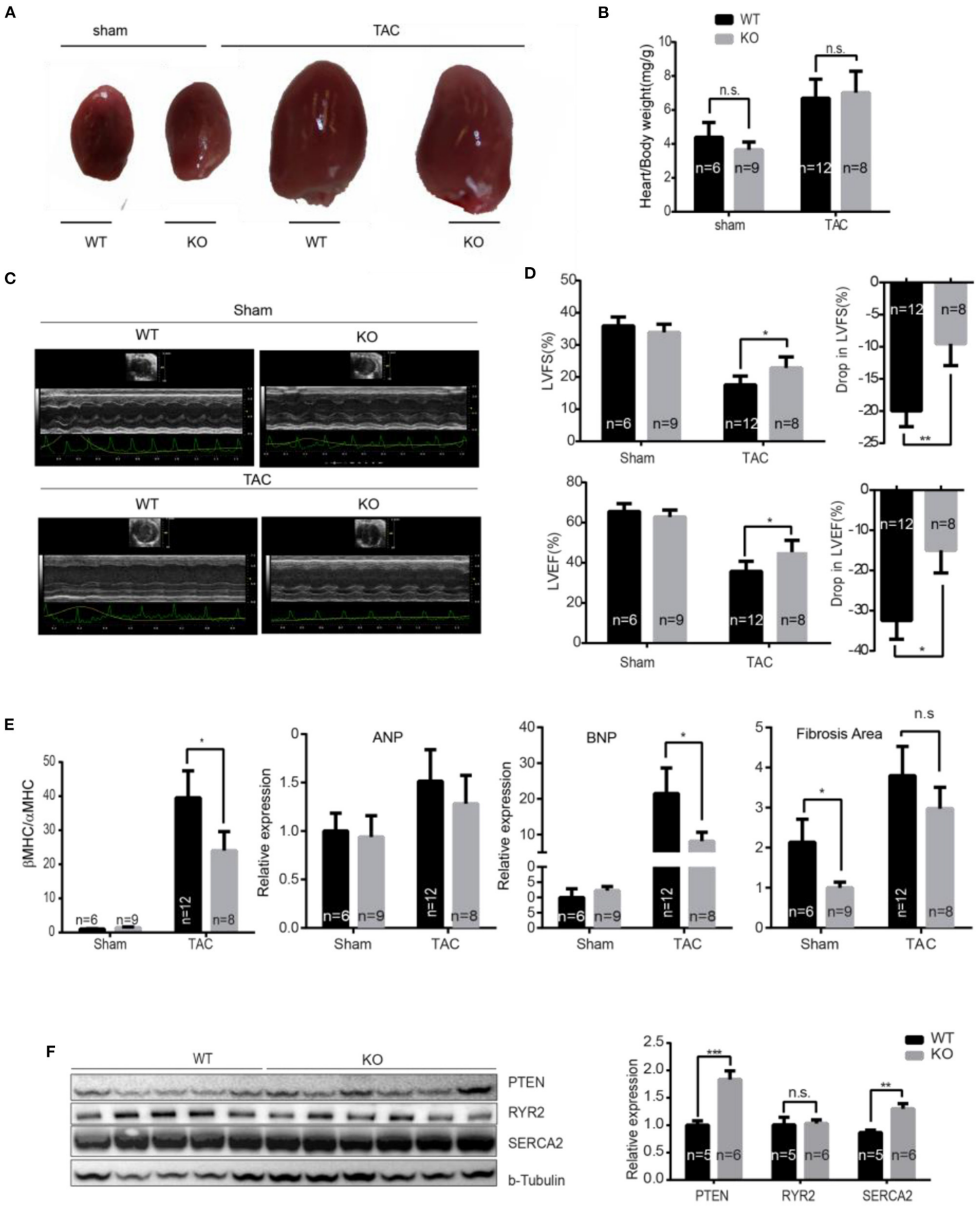
miR-132/212 KO mice are more resistant to pressure overload-induced loss of contractility. **(A)** Representative images of explanted WT and KO mice hearts 7 weeks after surgery. **(B)** Gravimetric analysis of heart weight of WT and KO mice 7 weeks after sham or TAC surgery. **(C)** Representative images of cardiac function assessment of WT and KO mice 7 weeks after sham or TAC surgery by echocardiography. **(D)** cardiac function assessment of WT and KO mice 7 weeks after sham or TAC surgery by Doppler echocardiography and the drop of cardiac function WT and KO mice before and after 7 weeks TAC. **(E)** Expression of cardiac stress marker Anp, Bnp, and βMHC/αMHC ratio and quantified fibrotic area by Picrosirius Red staining. Sham WT (*n* = 6) KO (*n* = 9), TAC WT (*n* = 12) KO (*n* = 8). **(F)** The expression of PTEN, Ryr2, and Serca2 in WT and KO mice 7 weeks after TAC detected by Western Blot and the quantification TAC WT (*n* = 5) KO (*n* = 6). *p < 0.05, **p < 0.01, ***p < 0.001. n.s indicates not statistical significantly different.

To assess the role of miR-132/212 on calcium handling and subsequent cardiac contractility during pathological heart failure progression, we performed TAC on miR-132/212 KO mice and wildtype control littermates. Seven weeks after TAC induction, an increase in heart/body weight ratio was observed but we did not observe a difference between KO and WT mice (Figures 4A,B). Interestingly, by echocardiography analysis, we observed a better-preserved cardiac function in KO mice (Supplementary Table 3), as indicated by better preserved Left Ventricular Fractional Shortening (LVFS) and preserved LV Ejection Fraction (LVEF) at 7 weeks after TAC (Figures 4C,D). Also, KO hearts showed lower expression of cardiac damage markers Bnp and a lower βMHC/αMHC ratio, which is indicative of a less diseased phenotype (Figure 4E). A non-significant trend toward lower Anp and fibrotic remodeling in KO hearts was observed (Figure 4E). In line with their better contractile function, KO mice showed a higher expression of SERCA2 and PTEN but not of Ryr2 (Figure 4F). These results suggest that animals without miR-132/212 show more resistant to pressure overload-induced heart failure, probably due to the absence of miR-132/212-mediated suppression of SERCA2 and PTEN.

### Expression of miR-132/212 and Their Targets in the Failing Human Heart

**Figure 5:**
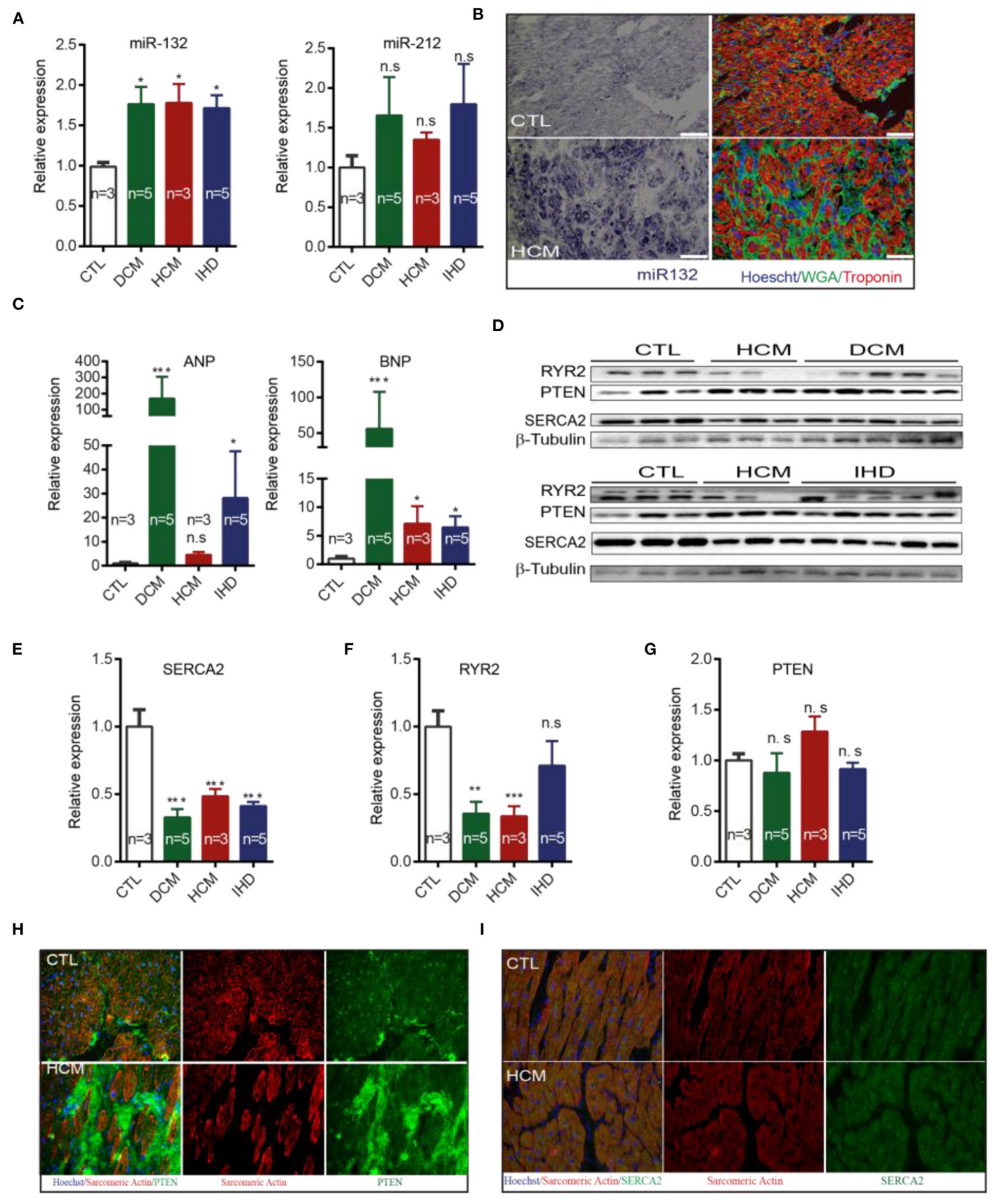
Expression of miR-132/212 and their targets in heart failure patients. **(A)** The relative expression of miR-132 and miR-212 in the left ventricle from healthy control, DCM, HCM and IHD heart failure patients. RNU6 is used as the internal control. **(B)** *in situ* detection of miR-132 (in purple-blue) in healthy controls and HCM patients; immunofluorescent staining for WGA for extracellular matrix in green, Troponin I for viable cardiomyocytes in red, nuclei in blue. **(C)** The relative expression of ANP and BNP mRNA in the left ventricle from healthy control, DCM, HCM, and IHD heart failure patients. GAPDH is used as the internal control. **(D)** The relative expression of RYR2, PTEN, and SERCA2 protein in left ventricle from healthy control, DCM, HCM, and IHD heart failure patients as detected by Western Blot. β-Tubulin is used as the internal control. **(E)** The relative expression of SERCA2a protein quantified from **(D)** and mRNA measured by qPCR in the left ventricle from healthy control, DCM, HCM, and IHD heart failure patients. GAPDH is used as the internal control. **(F,G)** Quantification of total endogenous RYR2 and PTEN in **(D)** corrected by β-Tubulin. **(H,I)** Representative image of immunofluorescent detection of PTEN and SERCA2 protein in health control and HCM heart failure patient left ventricle. Sarcomere Actinin is shown in red, PTEN in green and nuclei in blue. DCM, dilated cardiomyopathy; HCM, hypertrophic cardiomyopathy; IHD, ischemic heart disease. *p < 0.05,**p < 0.01, ***p < 0.001. n.s indicates not statistically significant different. Scalebar = 50 μm.

We further investigated whether miR-132/212 and their associated targets are similarly dysregulated in failing human hearts as in the mouse. First, we detected increased RNA expression levels of the mir-132 in failing hearts from dilated cardiomyopathy (DCM), hypertrophic cardiomyopathy (HCM) and ischemic cardiomyopathy (IHD) patients, as compared to the left ventricular free wall of healthy controls (Figure 5A). A similar pattern was also observed for miR-212, though differences did not reach significance due to larger variations (Figure 5A). By using miRNA *in situ* hybridization on LV sections of healthy hearts and HCM patients, we observed a larger cross-sectional diameter of the cardiomyocytes with a significantly increased presence of miR-132 in the HCM patients (Figure 5B).

As expected, DCM, HCM, and IHD patients display higher expression of the cardiac damage markers ANP and BNP when compared with healthy controls (Figure 5C). Only HCM patients failed to reach significance in ANP expression (Figure 5C). We subsequently examined the expression of the targets of miR-132/212 in end-stage heart failure patients. We observed that SERCA2 is down-regulated in DCM, HCM, and IHD patients at protein levels (Figures 5D,E,I), which is very common in the failing heart (3, 4). RyR2 was downreguated in DCM, HCM but not IHD patients (Figure 5F). These results suggest that SERCA2a could be a target of miR-132/miR-212 in the failing human heart. Western blotting did not confirm the downregulation of total PTEN in heart failure patients (Figures 5D,G). However, immunofluorescent staining showed reduced PTEN expression levels in cardiomyocytes, while PTEN appeared to be upregulated in other non-cardiomyocyte regions, indicating that PTEN might be playing a role in other cell types as well (Figure 5H).

## Discussion

**Figure 6:**
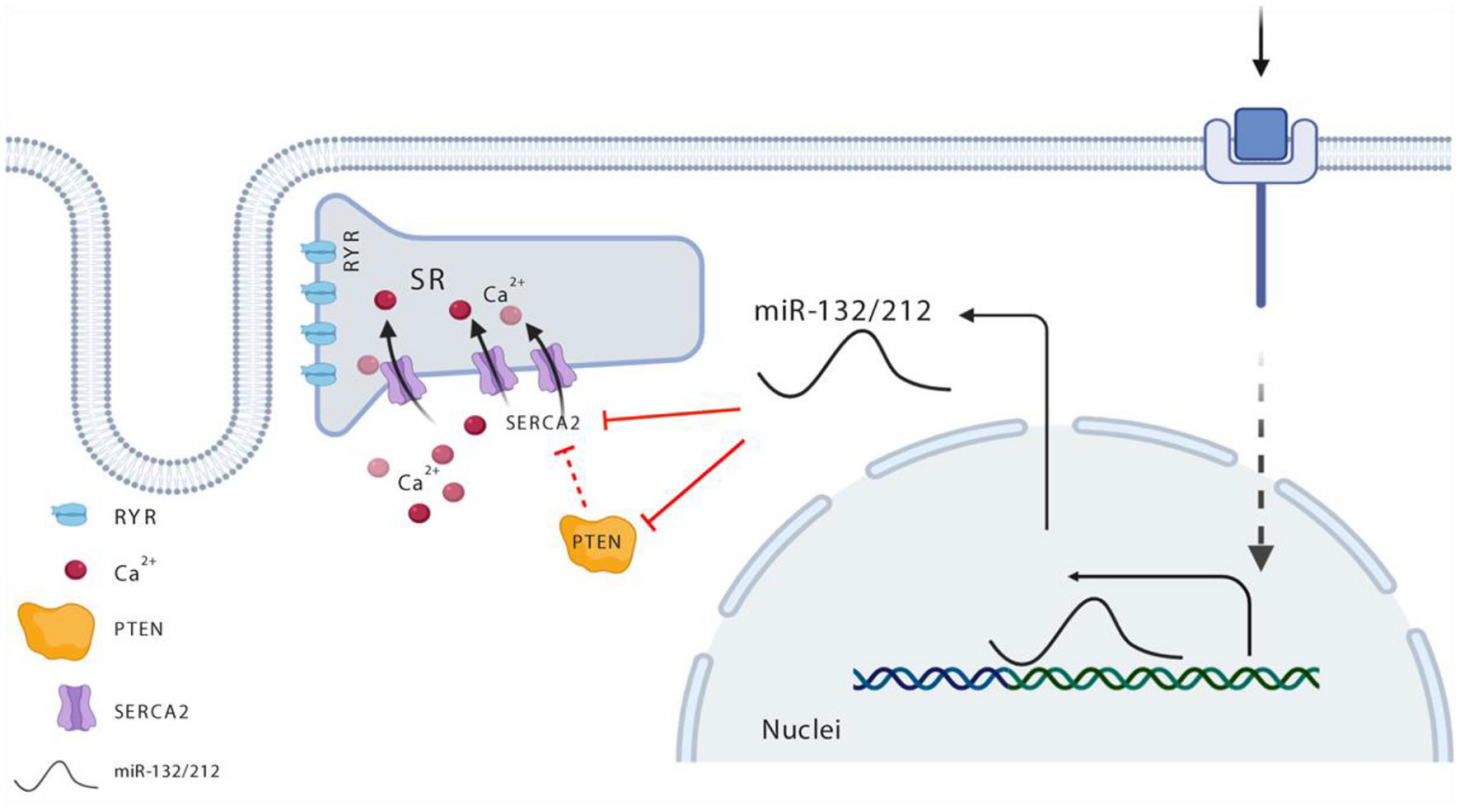
Schematic view for miR-132/212 regulation of cardiac contractility in the heart. Maintenance of the calcium gradient—namely, high calcium in the systolic phase and extremely low calcium in the diastolic phase—is key in the regulation of cardiomyocytes contractility, and is well-balanced and controlled *via* SERCA. miR-132/212 regulated SERCA directly as indicated with solid red line and indirectly as indicated with the broken red line *via* two paths, namely, it regulates the expression of SERCA2, or it regulated the activity of SERCA2 *via* PTEN. In heart failure situation, elevated miR-132/212 causes decreased cardiac contractility either by inhibiting the expression of SERCA2 or by lowering SERCA2 pump activity indirectly.

The upregulation of miR-132/212 in the stressed or failing hearts has been repeatedly observed, including human heart failure and mouse following pressure overload (11, 13, 14). We confirmed the upregulation of miR-132/212 in different categories of end-stage heart failure patients and cardiac hypertrophic mouse models. But how this upregulation is regulated is still unclear. Recently, four CREB (cAMP-response element-binding) sites have been identified within the miR-132/212 genomic loci (29). It is possible that stress, hormones or growth factors such as Ca^2+^, cAMP, or TGFβ induces CREB activation and thereby activates many downstream transcripts, including miR-132/212 (30, 31). Consistent with this hypothesis, studies show *in vitro* that miR-132/212 expression goes up upon stimulating the βAR (adrenergic receptor) pathway *via* βAR agonists (e.g., PE, Angiotensin II) (13). Since activation of the βAR pathway is a common mechanism with enhanced cardiac contractility, it is plausible that an over-stimulated βAR pathway could drive expression of miR-132/212 as a feedback control system, thereby suppressing contractility as a consequence of chronic βAR stimulation (Figure 6).

The upregulation of miR-132 in the failing heart is mainly in cardiomyocytes. Using miRNA *in situ* hybridization, we were able to provide additional details regarding the location of the expression of miR-132. We did detect the expression of miR-132 in other cell types, though expression level was relatively low, suggesting a secondary role in other cell types (32–34).

The upregulation of miR-132/212 found in failing heart cardiomyocytes might be detrimental to contractile function *in vivo*. We show that miR132 or miR212 overexpression can prolong calcium decay in RNCM. On the other hand, cardiomyocytes isolated from miR-132/212 KO mice displayed enhanced contractile capability and these KO mice display better preservation of cardiac function under chronic pressure overload stress. These loss-of-function studies suggest that blocking the upregulation of miR-132/212 could be beneficial in both maintaining cardiac contractile function and slowing the progression toward heart failure.

miR-132/212 modulates cardiomyocyte contractility, at least in part, through their regulation of SERCA2a. This is in line with previous findings that miR-132/212 can suppress GFP expression in the SERCA2a 3′-UTR reporter (12) and overexpression mir-132 in rat cardiomyocytes results in down-regulation of proteins enriched in contractile function (35). Three nucleic acid mutations within seed sequence could reverse the suppression effect. Furthermore, endogenous SERCA2a levels declined after the transfection of miR132 or miR212 in neonatal rat cardiomyocytes, and miR-132/212 KO mice showed higher levels of SERCA2a protein than wildtype controls, both in normal and stressed hearts. These results suggest that it might be a promising strategy to increase SERCA2a expression through the removal of miR-132/212 family (12).

Additionally, we identified PTEN as a direct target of miR-132/212 (36), suggesting that it might also mediate the effect of the miRNA on cardiomyocyte contractility. Loss of PTEN in cardiomyocytes results in a dramatic decrease in contractility (37, 38). Here, we demonstrated that knockdown of PTEN or overexpression of miR-132 or -212 both can significant slowdown the calcium decay in cardiomyocytes. We observed that overexpression of miR132 or miR212 resulted in the down-regulation of PTEN expression in cardiomyocytes. In line with this, miR-132/212 KO mice showed upregulated PTEN expression. However, in the failing human hearts, while the expression of miR-132/212 goes up, the expression of PTEN does not alter. This discrepancy of PTEN between mice and human patients might be explained by the location of PTEN expression shown by immunofluorescent staining. The upregulated PTEN expression in the non-cardiomyocytes fibrotic region, which might be more enriched in end stage patients than that of TAC mice heart, whereas the microRNAs are primarily upregulated in cardiomyocytes. Nevertheless, the underlying mechanism of how PTEN regulates Calcium kinetics still has to be further explored.

Our results are following most of the phenotypes observed in antimiR-132 treated TAC animals as well miR132/212 KO mice, in which these two miRs regulated cardiac hypertrophy and autophage by directly targeting FoxO3 and more resistant to pressure overload induced heart failure (13). We observed better cardiac function preservation after TAC in miR-132/212 KO mice, including less fibrosis, better ejection fraction and fraction shortening. We did not detect a significant difference in the heart/body weight ratio, although we did observe the better functional autophagic response in the KO mice and overexpression of miR132 or miR212 in RNCM induced hypertrophy (data is not shown). Our 7 weeks follow-up might cause such a discrepancy as the previous study terminated their mice at 3 weeks post-TAC. Here, we report that calcium dynamics are directly regulated by this miR-132/212.

Our results suggest that the upregulation of miR-132/212 in the failing heart may impair cardiac contractile function and accelerate the progression of heart failure. The pharmaceutical inhibition of miR-132/212 may, therefore, be a promising therapeutic approach to preserve cardiac function and slow down the progression of heart failure in patients.

## Data Availability Statement

The original contributions presented in the study are included in the article/Supplementary Material, further inquiries can be directed to the corresponding author/s.

## Ethics Statement

The animal study was reviewed and approved by the Animal Ethical Experimentation Committee (DEC. 2013.II.02.019, Utrecht University).

## Author Contributions

ZL and JS conceived this study. ZL, CW, HA, JD, AM, AR-M, DK, and MH performed experiment and analyzed data. MM, RW, JV, PD, and JS supervised this study. ZL, CW, MM, JX, PD, and JS wrote the paper. All authors contributed to the article and approved the submitted version.

## Conflict of Interest

The authors declare that the research was conducted in the absence of any commercial or financial relationships that could be construed as a potential conflict of interest.

## References

[1] Bers DM. Cardiac excitation–contraction coupling. Nature. (2002) 415:198–205. 10.1038/415198a 11805843

[2] Frank KF Bölck B Erdmann E Schwinger RH. Sarcoplasmic reticulum Ca2+-ATPase modulates cardiac contraction and relaxation. Cardiovasc Res. (2003) 57:20–7. 10.1016/S0008-6363(02)00694-6 12504810

[3] Borlak J Thum T. Hallmarks of ion channel gene expression in end-stage heart failure. FASEB J. (2003) 17:1592–608. 10.1096/fj.02-0889com 12958166

[4] Meyer M Schillinger W Pieske B Holubarsch C Heilmann C Posival H. Alterations of sarcoplasmic reticulum proteins in failing human dilated cardiomyopathy. Circulation. (1995) 92:778–84. 10.1161/01.CIR.92.4.778 7641356

[5] Niwano K Arai M Koitabashi N Watanabe A Ikeda Y Miyoshi H. Lentiviral vector-mediated SERCA2 gene transfer protects against heart failure and left ventricular remodeling after myocardial infarction in rats. Mol Therapy. (2008) 16:1026–32. 10.1038/mt.2008.61 18388909

[6] Jessup M Greenberg B Mancini D Cappola T Pauly DF Jaski B. Calcium upregulation by percutaneous administration of gene therapy in cardiac disease, calcium upregulation by percutaneous administration of gene therapy in cardiac disease (CUPID): a phase 2 trial of intracoronary gene therapy of sarcoplasmic reticulum Ca2+-ATPase in patients with advanced heart failure. Circulation. (2011) 124:304–13. 10.1161/CIRCULATIONAHA.111.022889 21709064 PMC5843948

[7] Greenberg B Yaroshinsky A Zsebo KM Butler J Felker GM Voors AA. Design of a phase 2b trial of intracoronary administration of AAV1/SERCA2a in patients with advanced heart failure: the CUPID 2 trial (calcium up-regulation by percutaneous administration of gene therapy in cardiac disease phase 2b). JACC. Heart Failure. (2014) 2:84–92. 10.1016/j.jchf.2013.09.008 24622121

[8] Ziolo MT Martin JL Bossuyt J Bers DM Pogwizd SM. Adenoviral gene transfer of mutant phospholamban rescues contractile dysfunction in failing rabbit myocytes with relatively preserved SERCA function. Circulation Res. (2005) 96:815–7. 10.1161/01.RES.0000163981.97262.3b 15790952

[9] van Rooij E Sutherland LB Qi X Richardson JA Hill J Olson EN. Control of stress-dependent cardiac growth and gene expression by a microRNA. Science. (2007) 316:575–9. 10.1126/science.1139089 17379774

[10] Kwekkeboom RF Lei Z Doevendans PA Musters RJ Sluijter JP. Targeted delivery of miRNA therapeutics for cardiovascular diseases: opportunities and challenges. Clin Sci. (2014) 127:351–65. 10.1042/CS20140005 24895056

[11] Thum T Galuppo P Wolf C Fiedler J Kneitz S van Laake LW. MicroRNAs in the human heart: a clue to fetal gene reprogramming in heart failure. Circulation. (2007) 116:258–67. 10.1161/CIRCULATIONAHA.107.687947 17606841

[12] Wahlquist C Jeong D Rojas-Munoz A Kho C Lee A Mitsuyama S. Inhibition of miR-25 improves cardiac contractility in the failing heart. Nature. (2014) 508:531–5. 10.1038/nature13073 24670661 PMC4131725

[13] Ucar A Gupta SK Fiedler J Erikci E Kardasinski M Batkai S. The miRNA-212/132 family regulates both cardiac hypertrophy and cardiomyocyte autophagy. Nat Commun. (2012) 3:1078. 10.1038/ncomms2090 23011132 PMC3657998

[14] van Rooij E Sutherland LB Thatcher JE DiMaio JM Naseem RH Marshall WS. Dysregulation of microRNAs after myocardial infarction reveals a role of miR-29 in cardiac fibrosis. Proc Natl Acad Sci USA. (2008) 105:13027–32. 10.1073/pnas.0805038105 18723672 PMC2529064

[15] Kayo H Kiga K Fukuda-Yuzawa Y Hedlund S Murakami K De La Rosa-Velazquez IA. miR-212 and miR-132 are dispensable for mouse mammary gland development. Nat Genet. (2014) 46:802–4. 10.1038/ng.2990 25070796

[16] Lei Z Fang J Deddens JC Metz CH van Eeuwijk EC El Azzouzi H. Loss of miR-132/212 has no long-term beneficial effect on cardiac function after permanent coronary occlusion in mice. Front Physiol. (2020) 11:590. 10.3389/fphys.2020.00590 32612537 PMC7309700

[17] El Azzouzi H Leptidis S Dirkx E Hoeks J van Bree B Brand K. The hypoxia-inducible microRNA cluster miR-199a ~214 targets myocardial PPARdelta and impairs mitochondrial fatty acid oxidation. Cell Metabol. (2013) 18:341–54. 10.1016/j.cmet.2013.08.009 24011070

[18] Lei Z van Mil A Xiao J Metz CHG van Eeuwijk ECM Doevendans PA. MMISH: Multicolor microRNA *in situ* hybridization for paraffin embedded samples. Biotechnol Rep. (2018) 18:e00255. 10.1016/j.btre.2018.e00255 29876304 PMC5989586

[19] Crowley SD Gurley SB Herrera MJ Ruiz P Griffiths R Kumar AP. Angiotensin II causes hypertension and cardiac hypertrophy through its receptors in the kidney. Proc Natl Acad Sci USA. (2006) 103:17985–90. 10.1073/pnas.0605545103 17090678 PMC1693859

[20] Chu G Carr AN Young KB Lester JW Yatani A Sanbe A. Enhanced myocyte contractility and Ca2+ handling in a calcineurin transgenic model of heart failure. Cardiovasc Res. (2002) 54:105–16. 10.1016/S0008-6363(02)00230-4 12062367

[21] Arber S Hunter JJ Ross J Jr Hongo M Sansig G Borg J. MLP-deficient mice exhibit a disruption of cardiac cytoarchitectural organization, dilated cardiomyopathy, and heart failure. Cell. (1997) 88:393–403. 10.1016/S0092-8674(00)81878-4 9039266

[22] Sluijter JP van Mil A van Vliet P Metz CH Liu J Doevendans PA. MicroRNA-1 and -499 regulate differentiation and proliferation in human-derived cardiomyocyte progenitor cells. Arterioscler Thrombosis Vasc Biol. (2010) 30:859–68. 10.1161/ATVBAHA.109.197434 20081117

[23] Cerignoli F Charlot D Whittaker R Ingermanson R Gehalot P Savchenko A. High throughput measurement of Ca2+ dynamics for drug risk assessment in human stem cell-derived cardiomyocytes by kinetic image cytometry. J Pharmacol Toxicol Methods. (2012) 66:246–56. 10.1016/j.vascn.2012.08.167 22926323 PMC3667588

[24] Agarwal V Bell GW Nam J-W Bartel DP. Predicting effective microRNA target sites in mammalian mRNAs. Elife. (2015) 4:e05005. 10.7554/eLife.05005 26267216 PMC4532895

[25] van Mil A Grundmann S Goumans MJ Lei Z Oerlemans MI Jaksani S. MicroRNA-214 inhibits angiogenesis by targeting Quaking and reducing angiogenic growth factor release. Cardiovasc Res. (2012) 93:655–65. 10.1093/cvr/cvs003 22227154

[26] Whittaker R Cerignoli F Ingermanson R Towart R Gallacher DJ Mercola M. Simultaneous recording of action potentials and calcium transients from stem cell-derived cardiomyocytes: applications for cardiotoxicity testing. J Pharmacol Toxicol Methods. (2013) 68:e47. 10.1016/j.vascn.2013.01.170

[27] Karolchik D Baertsch R Diekhans M Furey TS Hinrichs A Lu Y. The UCSC genome browser database. Nucl Acids Res. (2003) 31:51–4. 10.1093/nar/gkg129 12519945 PMC165576

[28] Jin W Reddy MA Chen Z Putta S Lanting L Kato M. Small RNA sequencing reveals microRNAs that modulate angiotensin II effects in vascular smooth muscle cells. J Biol Chem. (2012) 287:15672–83. 10.1074/jbc.M111.322669 22431733 PMC3346099

[29] Remenyi J Hunter C Cole C Ando H Impey S Monk C. Regulation of the miR-212/132 locus by MSK1 and CREB in response to neurotrophins. Biochem J. (2010) 428:281–91. 10.1042/BJ20100024 20307261

[30] Shaywitz AJ Greenberg ME. CREB: a stimulus-induced transcription factor activated by a diverse array of extracellular signals. Ann Rev Biochem. (1999) 68:821–61. 10.1146/annurev.biochem.68.1.821 10872467

[31] Mayr B Montminy M. Transcriptional regulation by the phosphorylation-dependent factor CREB. Nat Rev Mol Cell Biol. (2001) 2:599–609. 10.1038/35085068 11483993

[32] Lei Z Mil A Brandt MM Grundmann S Hoefer I Smits M. MicroRNA-132/212 family enhances arteriogenesis after hindlimb ischaemia through modulation of the Ras-MAPK pathway. J Cell Mol Med. (2015) 19:1994–2005. 10.1111/jcmm.12586 25945589 PMC4549050

[33] Eskildsen TV Jeppesen PL Schneider M Nossent AY Sandberg MB Hansen PB. Angiotensin II regulates microRNA-132/-212 in hypertensive rats and humans. Int J Mol Sci. (2013) 14:11190–207. 10.3390/ijms140611190 23712358 PMC3709727

[34] Kumarswamy R Volkmann I Beermann J Napp LC Jabs O Bhayadia R. Vascular importance of the miR-212/132 cluster. Eur Heart J. (2014) 35:3224–31. 10.1093/eurheartj/ehu344 25217442

[35] Foinquinos A Batkai S Genschel C Viereck J Rump S Gyongyosi M. Preclinical development of a miR-132 inhibitor for heart failure treatment. Nat Commun. (2020) 11:633. 10.1038/s41467-020-14349-2 32005803 PMC6994493

[36] Lei Z Klasson TD Brandt MM van de Hoek G Logister I Cheng C. Control of angiogenesis *via* a VHL/miR-212/132 axis. Cells. (2020) 9:1017. 10.3390/cells9041017 32325871 PMC7226144

[37] Crackower MA Oudit GY Kozieradzki I Sarao R Sun H Sasaki T. Regulation of myocardial contractility and cell size by distinct PI3K-PTEN signaling pathways. Cell. (2002) 110:737–49. 10.1016/S0092-8674(02)00969-8 12297047

[38] Ruan H Li J Ren S Gao J Li G Kim R. Inducible and cardiac specific PTEN inactivation protects ischemia/reperfusion injury. J Mol Cell Cardiol. (2009) 46:193–200. 10.1016/j.yjmcc.2008.10.021 19038262

