# Supplementary material for: miR-132/212 Impairs Cardiomyocytes Contractility in the Failing Heart by Suppressing SERCA2a
Source: Front Cardiovasc Med. 2021 Mar 19;8:592362. doi: 10.3389/fcvm.2021.592362 (PMC8017124; doi:10.3389/fcvm.2021.592362)
Supplement: Supplementary file 1 [file Data_Sheet_1.docx]

**Supplementary material**


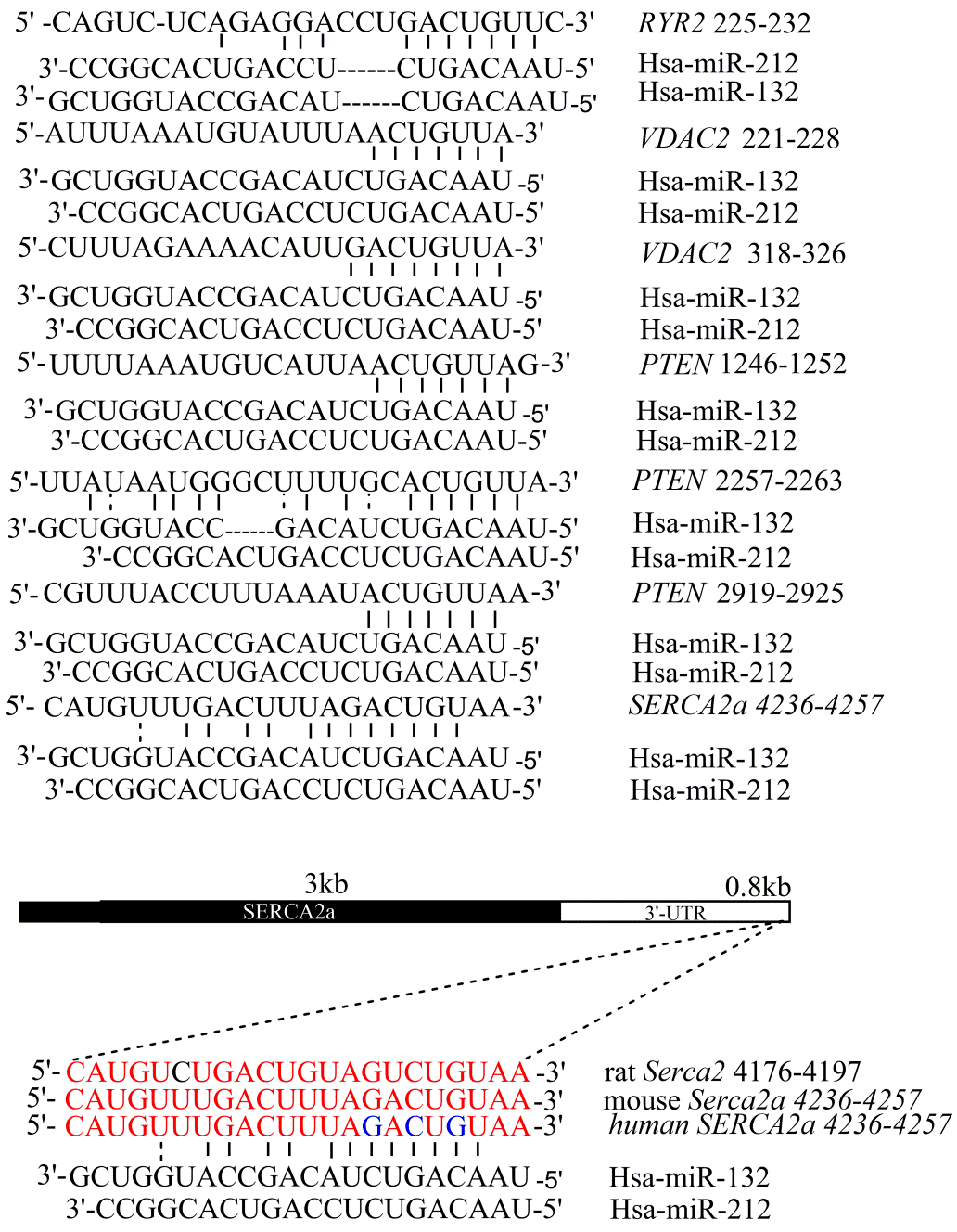


A

Supplementary Figure 1. Predicted miR-132/212 targeting site on 3'-UTR of Pten, Ryr2 and VDAC2..

Figure 2. The relative expression of mir-132 and mir-212 after transfection of pre-mir-132 or -212 and antimir-132, or 212.

**
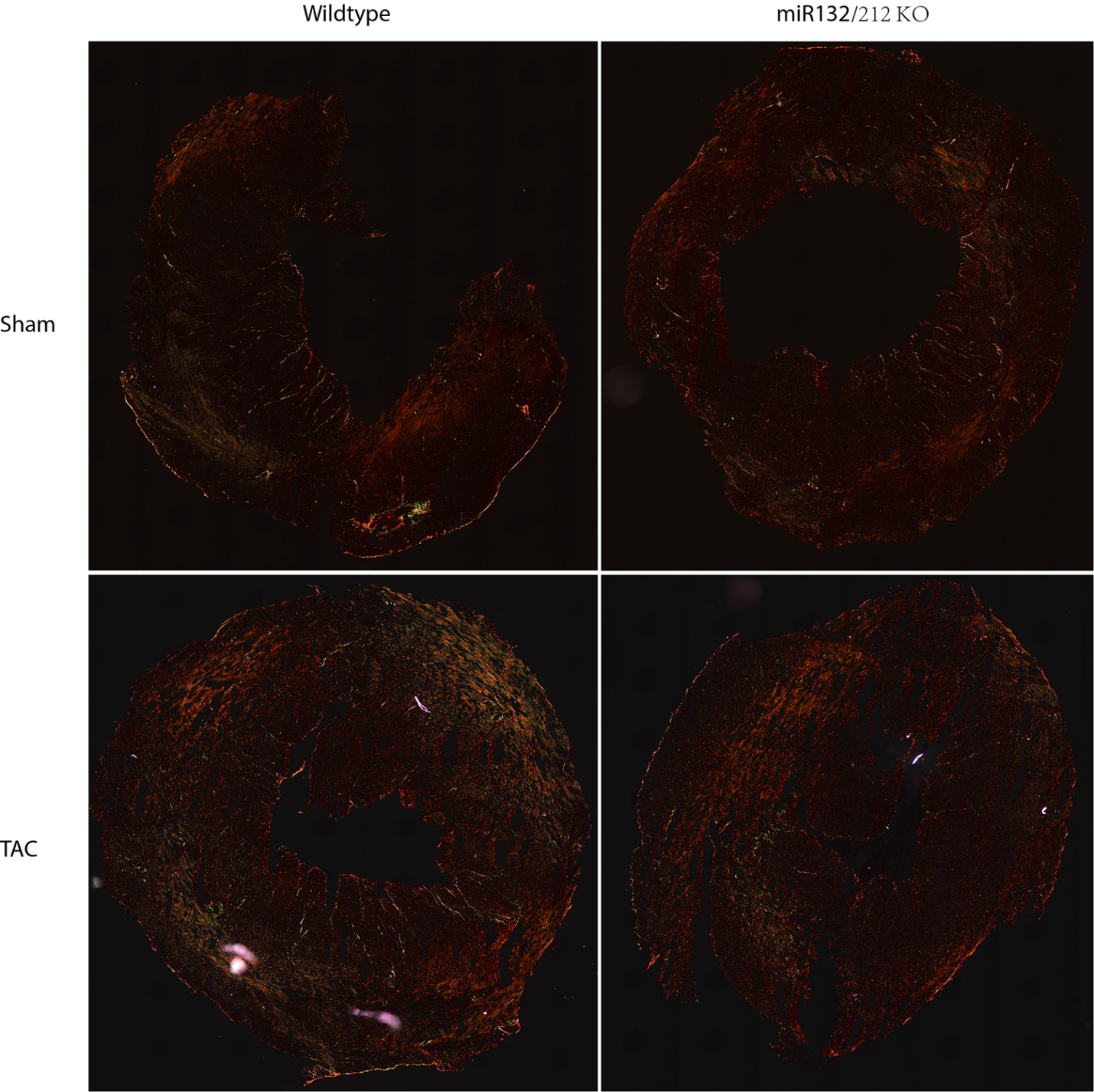
Supplementary Figure 3. Representative images of picrosirius red staining in Sham and TAC operated mouse heart.**

| **Primer name** | **Sequence(5'-3’)** | **Species** | **Application** |
| --- | --- | --- | --- |
| Mir132-KO-GT-P1 | ATCCTTTCAAGAAAGTGGGGAGA | mouse | genotyping |
| Mir132-KO-GT-P2 | TCTGAGGAGGATGTTCAGACAC | mouse | genotyping |
| Gapdh_F | GGCATGGACTGTGGTCATGA | mouse | qPCR |
| Gapdh_R | TTCACCACCATGGAGAAGGC | mouse | qPCR |
| Serca2a_3'utr_SpeI | ATCGACTAGTCAATACTGGAGTAACCGCTTCCT | mouse | luciferase assay |
| Serca2a_3'utr_HindIII | ACTGAAGCTTCATAGAATAGATTTATTTACCTGAA | mouse | luciferase assay |
| Anp_F1 | CCTGTGTACAGTGCGGTGTC | mouse | qPCR |
| Anp_R1 | CCTAGAAGCACTGCCGTCTC | mouse | qPCR |
| Bnp_F1 | GTTCTTTTGTGAGGCCTTGG | mouse | qPCR |
| Bnp_R1 | CTGAAGGTGCTGTCCCAGAT | mouse | qPCR |
| αMHC_F1 | GGTCCACATTCTTCAGGATTCTC | mouse | qPCR |
| αMHC_R1 | GCGTTCCTTCTCTGACTTTCG | mouse | qPCR |
| βMHC_F1 | CAGGCCTGTAGAAGAGCTGTACTC | mouse | qPCR |
| βMHC_R1 | TCTCCTGCTGTTTCCTTACTTGCT | mouse | qPCR |
| PTEN_F1 | TGGATTCGACTTAGACTTGACCT | mouse | qPCR |
| PTEN_R1 | GCGGTGTCATAATGTCTCTCAG | mouse | qPCR |
| Q5_SERCA2a_3UTR_Mut_F | GTCTAAATAGAGATCAGTTTGTTTCTTTC | mouse | luciferase assay |
| Q5_SERCA2a_3UTR_Mut_R | TGTAAAGTCAAACATGCGCAGTG | mouse | luciferase assay |
| Serca2a(b)_F1 | TGGAACCTTTGCCGCTCATT | mouse | qPCR |
| Serca2a_R1 | CGGTTACTCCAGTATTGCGG | mouse | qPCR |
| Serca2b_R1 | CTGCACACACTCTTTACCGG | mouse | qPCR |

**Table 1 Primers and oligo used in this study**

**Table 2 reagents used in this study**

| **Product name** | **Company** | **Catology No.** | **Dilution** | **Application** |
| --- | --- | --- | --- | --- |
| anti-PTEN (138G6) antibody | Cell signaling | 9559S | 1/1000 | WB |
| anti-RyR2 antibody | Thermo Scientific | MA3-925 | 1/1000 | WB |
| anti-GAPDH antibody | Cell signaling | #2118S | 1/1000 | WB |
| anti-Vimentin | Abcam | ab45939 | 1/1600 | WB |
| anti-Dig-AP Fab fragment | Roche | 11093274910 | 1/1500 | ISH |
| β-Tubulin (9F3) | Cell signaling | #6181 | 1/1000 | WB |
| BCIP/NBT | DAKO | K0598 | 1/1000 | ISH |
| anti-Vimentin antibody V9 | Sigma | V6630 | 1/1000 | WB |
| β-actin antibody AC-15 | Sigma | A5441 | 1/1000 | WB |
| anti-SERCA2 [2A7-A1] | Abcam | ab2861 | 1/1000 | IF |
| anti-SERCA2 (N-19) | Santa Crutz | sc-8095 | 1/1000 | WB |
| Lectin BS-I | Sigma | L2895 | 1/200 | IF |
| WGA | Sigma | L4895 | 1/500 | IF |

WB: Western blot; ISH: in situ hybridization; IF: immunfluorescence staining

**Table 3 Echocardiography assessment of cardiac function**

|  | 7 weeks Sham |  | 7 weeks TAC |  |
| --- | --- | --- | --- | --- |
|  | WT(n=6) | KO(n=9) | WT(n=12) | KO(n=8) |
| Heart_Rate(BMP) | 548.9±11.5 | 442.8±12.9** | 467.3±14.2 | 463.5±13.9 |
| Diameter_systolic(mm) | 2.6±0.2 | 2.4±0.2 | 3.4±0.2 | 3.0±0.3 |
| Diameter_diastolic(mm) | 4.0±0.1 | 3.7±0.2 | 4.0±0.2 | 3.9±0.2 |
| Volume_systolic(a.u) | 25.3±4.5 | 24.0±4.7 | 49.4±6.4 | 38.8±7.4 |
| Volume_diastolic(a.u) | 70.8±5.8 | 61.6±7.8 | 73.3±6.5 | 66.4±7.8 |
| Stroke_volume(a.u) | 45.5±2.7 | 37.6±4.6 | 23.9±2.6 | 27.6±2.1 |
| Ejection_Fraction(%) | 65.6±3.9 | 62.5±3.5 | 35.7±5.0 | 45.4±5.8* |
| Fraction_shortening(%) | 35.9±2.8 | 33.6±2.5 | 17.5±2.8 | 22.8±3.4* |
| Cardiac_Output (a.u.) | 25.0±1.6 | 16.6±2.0* | 10.9±1.0 | 12.8±1.0 |
| IVS_d(mm) | 0.99±0.05 | 1.14±0.05 | 1.03±0.04 | 0.99±0.03 |
| IVS_S(mm) | 1.42±0.08 | 1.56±0.05 | 1.36±0.06 | 1.25±0.07 |
| LVID_d(mm) | 3.82±0.21 | 3.45±0.10 | 3.71±0.13 | 4.03±0.21 |
| LVID_s(mm) | 2.61±0.22 | 2.42±0.09 | 3.05±0.16 | 3.38±0.24 |
| LVPW_d(mm) | 1.17±0.11 | 1.12±0.07 | 1.22±0.08 | 0.98±0.04 |
| LVPW_s(mm) | 1.48±0.13 | 1.45±0.08 | 1.34±0.07 | 1.19±0.06 |
| LV_mass(mg) | 166.56±11.96 | 152.52±6.53 | 170.96±11.96 | 159.61±11.37 |
| LV_mass_corrected(mg) | 133.25±9.58 | 122.02±5.22 | 136.77±9.57 | 127.69±9.09 |
